# Savvy and woke: Gender, digital profile, social media competence, and political participation in gender issues among young Filipino netizens

**DOI:** 10.3389/fsoc.2022.966878

**Published:** 2022-07-28

**Authors:** Julienne Celina Sicat Dayrit, Blulean Terosa Albao, Jerome Visperas Cleofas

**Affiliations:** ^1^Department of Sociology and Behavioral Sciences, De La Salle University, Manila, Philippines; ^2^Department of Literature, De La Salle University, Manila, Philippines

**Keywords:** gender, political participation, social media, social media competence, youth activism

## Abstract

Social media has become a viable platform for political participation in issues related to gender, especially among the youth. Evidence suggests that gender and sexual identities, digital access, and skills foster political participation in social media. This study sought to determine the predictive relationship of gender, digital profile, and social media competence with social media political participation in gender issues (SMPP-GI) among young Filipino netizens through the lenses of social identity theory and resource model of political participation. A total of 1,090 college netizens aged 18–30 years old participated in this cross-sectional study. An online survey was used to collect data. The respondents reported low to moderate levels of SMPP-GI. Females and non-cisheterosexual respondents report higher scores in certain types of SMPP-GI. Respondents using more social media sites have higher levels of latent and counter engagement SMPP-GI. Among the four domains of social media competence, content generation significantly predicted all types of SMPP-GI, while content interpretation and anticipatory reflection were significantly linked with at least one type of engagement.

## Introduction

Like other aspects and events of life in the contemporary world, social movements toward social justice have also found their way into cyberspace. With around five billion people using the internet worldwide (Statista Research Department, 2021a), the pervasiveness of social media has shaped citizens' political participation regarding contemporary social issues (Murthy, 2018). We suspect that social media political participation (SMPP) would have increased during the time of the 2019 novel coronavirus (COVID-19) pandemic, when lockdowns, and social distancing restrictions have shifted most social interactions online, causing an increased user growth rate in social media across the globe in 2020 (Statista Research Department, 2021a). A wealth of research has indicated the positive influence of social media use on political participation (Dimitrova and Bystrom, 2013; Kim and Khang, 2014; Boulianne, 2015; Lee and Xenos, 2020), especially among the youth (Ida and Saud, 2020). In terms of focus and context of SMPP, studies have examined generalized forms of online political participation (Shehzad et al., 2021), and specific issues such as election (Dimitrova and Bystrom, 2013; Yang and DeHart, 2016), racism (Ince et al., 2017) and gender inequality (Quan-Haase et al., 2021).

Our present study specifically focuses on social media participation in gender issues (SMPP-GI) in the Philippines. Based on the 2020–2021 summary report on the state of Sustainable Development Goals (SDGs) related to gender equality, the country appears to be in a good position among its Southeast Asian (ASEAN) counterparts. The Philippines is among the countries with a lower proportion of women involved in domestic violence, child marriage, and gender disparities in public positions (U. N. Women, 2021). However, the current regime has been characterized by misogynistic posturing, chauvinist populism, and sexist remarks by President Rodrigo Duterte, which could potentially undermine developmental efforts to achieve a more gender-equal nation (Go, 2019; Parmanand, 2020). The Philippines dropped from the 5th spot in 2013 to 16th in 2020 against other countries in the Global Gender Gap Index (Mercurio, 2019). This may be attributed to the lack of prioritization of women, children, and gender policies during the Duterte regime (Abad, 2020).

Social media has become a viable platform for Filipinos in terms of political participation in gender issues. The Philippines has been dubbed the “Social Media Capital of the World,” where an average internet user spends almost 4 h per day on social media during the pandemic year 2020 (Ichimura, 2020). Around 30 percent of these users are within the youth bracket (Statista Research Department, 2021b). Feminist movements have emerged on social media such as #BabaeAko (I am a Woman), which was a push back against President Duterte's comment against a woman occupying the Chief Justice position (Haynes, 2018), and #HijaAko (I am a Daughter), which was a counter-movement against the prevalent victim-blaming of women who were raped and sexually harassed (Foundation for Media Alternatives, 2020). Likewise, lesbian, gay, bisexual, trans, queer, plus (LGBTQ+) movements have found an alternative space online, especially during the COVID-19 pandemic. Instead of in-person June Pride Marches in 2020 and 2021, LGBTQ+ groups have implemented social media-mediated campaigns, events, lectures, and performances for gender equality and LGBTQ+ rights (Adel, 2021).

Evidence has underscored the power of social media political participation in promoting offline political participation (Gil de Zúñiga et al., 2012; Strömbäck et al., 2018) and stimulating positive social change toward gender equality (Seibicke, 2017). Given the current status of gender and development in the Philippines, we assert the relevance of examining the factors influencing SMPP-GI among Filipino youth, who are currently underrepresented in this area of study. We appealed to the perspectives of social identity theory and resource model in identifying possible factors that influence SMPP-GI. This present study aims to test the predictive relationship of gender, digital profile, and social media competence, with social media political participation in gender issues among young Filipino netizens.

## Literature review and theoretical frameworks

### Conceptualizing social media political participation

Van Deth (2014) characterizes political participation as any of the following voluntary, non-professional activities: activities within the locus of politics, government, or state; activities that target politics, social problems, or communities; or non-political activities that have political motivations. A meta-analysis of earlier social-media-related studies (Boulianne, 2015) considered campaigning, protesting, civic engagement, and the combinations of any of the three as political participation. Other scholars argued that more passive behaviors such as slacktivism should be considered SMPP (Piat, 2019; Madison and Klang, 2020). In recent years, political participation on social media had been attributed to “wokeness,” which refers to awareness of and expressions against social injustices (Allen, 2020). Being woke (or “*mulat”* in Filipino) has been linked to positive (e.g., sociopolitical awareness; Caldera, 2018) and negative attributes (e.g., conflict-inducing and being un-Christian; Strachan, 2021). For this present study, we intend to cover more diverse forms of behaviors to signify SMPP, unlike many of the previous studies that examined only particular behaviors related to SMPP (Boulianne, 2015). Hence, we appeal to the social media political participation framework developed by Waeterloos et al. (2021), who forwarded a more encompassing understanding of SMPP, including passive and active forms of political engagement. Waeterloos et al. (2021) suggested four types of SMPP. First is *latent engagement*, which refers to the consumption of information on social or political issues, as demonstrated in previous studies (Gil de Zúñiga et al., 2012; Yamamoto and Nah, 2018). The second is *counter engagement*, which includes performing uncivil behaviors, such as misusing private information (George and Leidner, 2019) and negative commenting (Khan et al., 2019), for social and political causes. The third is *follower engagement*, which involves participation and promotion of the content and activities of other social media users, also coined as metavoicing (Albu and Etter, 2018; George and Leidner, 2019). Fourth is *expressive engagement*, which refers to the social media actions and posts that were instigated by users themselves—constructs commonly explored by recent research on gender-related SMPP (Ciszek, 2017; Quan-Haase et al., 2021). Taking these dimensions together, this framework for SMPP would be able to cater to both positive and negative aspects of wokeness.

### Social identity theory, gender, and SMPP

Social identity theory posits that an aspect of an individual's self-concept is constituted by one's membership in groups, and such identity motivates political behavior (Kalin and Sambanis, 2018). Social media provides affordances for political participation and activism such as page following, group membership, and hashtags that allow people to signify their social identity (Zappavigna, 2015; George and Leidner, 2019). Individuals desire to improve their group's standing in society by maintaining the group's highly valued attributes and/or fighting to rectify the group's negative image (Tajfel and Turner, 1979). The dominance of patriarchy and cisheterosexism in society breeds conditions that marginalize women and lesbian, gay, bisexual, trans, queer, and other non-cisheterosexual (LGBTQ+) identifying persons (Cravens, 2020; Fitzgerald and Grossman, 2020). These conditions can stimulate political participation among members of these oppressed gender categories, which in turn, enhance the group's collective identity, and strengthen the members' sense of personal identity (Vestergren et al., 2017; Cravens, 2020; Foster et al., 2021). Since our present study examines SMPP specific to gender issues, we chose gender as our first explanatory variable of interest. The current body of research on the gendered differences in online political participation points more to males (Vochocová et al., 2016; Quaranta and Dotti Sani, 2018; Ahmed and Madrid-Morales, 2021) than females (Yang and DeHart, 2016). However, the context of the political participation measured in these studies was not specific to gender-issues. Studies that did examine SMPP related to gender issues did not, however, attempt to quantitatively differentiate females from males, and/or cisheterosexual identities from LGBTQ+ counterparts (Jones and Brewster, 2017; Foster et al., 2021; Quan-Haase et al., 2021; Thompson and Turnbull-Dugarte, 2021). Our first research objective attempts to test how SMPP-GI is influenced by gender, which covers sex assigned at birth, sexual orientation, and gender identity. Hence, we hypothesize:

***H***_**1**_***:*** ***Females and respondents who identify as LGBTQ*****+** ***will demonstrate higher SMPP-GI***.

### Resource model, digital profile, social media competence and SMPP

The resource model of political participation states that the extent of political participation is shaped by one's available resources, which may include time, money, and other assets (Brady et al., 1995). In the era of new media, access to social media has become an invaluable resource for various forms of political participation. Access to tangible digital tools is a prerequisite to maximizing the affordances of social media to facilitate political participation. We coined these prerequisites to SMPP as ***digital profile***, which includes having active accounts on one or more social media sites (Effing et al., 2011; Abdu et al., 2017; Albu and Etter, 2018), ownership of one or more gadgets (Schradie, 2011; Kim et al., 2016; Lin and Chiang, 2017), and internet connection (Wang et al., 2018; Shehzad et al., 2021). For the second research objective of the study, we will determine how the respondents' digital profile influences their SMPP-GI. Therefore, we hypothesize:

***H***_**2**_***:*** ***Respondents who are active on more social media sites, own more gadgets, and*** ***have access to better internet quality will demonstrate higher SMPP-GI***.

The resource model also suggests that aside from tangible tools, skills are also resources that contribute to political participation (Brady et al., 1995). In the online realm, Internet skills have been indicated as facilitators of youth political participation (Wang et al., 2018). Various measures of social media ability have been linked to better political participation, such as social media self-efficacy (Omotayo and Folorunso, 2020; Hoffmann and Lutz, 2021), prosumption, and information literacy (Yamamoto and Nah, 2018; Tugtekin and Koc, 2020; Yamamoto et al., 2020) and creative expressions (Yu and Oh, 2018; Zhu et al., 2019). For this present study, we operationalized SMPP-related skills and abilities as ***social media competence*** (Zhu et al., 2020), which includes four domains, such as technical usability, content interpretation, content generation, and anticipatory reflection. These attributes are aspects of being what is colloquially known as “social media savvy” (Miller, 2018). For our third research objective, we will test the predictive relationship between social media competence and SMPP-GI. Hence, hypothesize:

1. ***H3: Respondents with higher social media competence will demonstrate*** ***higher SMPP-GI***.

## Methods

### Study design and participants

This study utilized a cross-sectional design. The target participants are Filipino netizens who are aged 18–30 years old, which is the bracket considered as “youth” by the National Youth Commission (2021). We were interested in college-based Filipino youth as the sample for this study because despite being a large sector of the population, they face cultural barriers to offline political participation in the country; hence, the viability of social media as their main platform for political engagement (International IDEA., 2020). A total of 1,090 conveniently sampled respondents qualified for the study. Majority of the respondents are 21 years old (μ = 20.108 ± 1.802), from North and Central Luzon (*n* = 550, 50.459%), and enrolled in STEM degree programs (*n* = 382, 35.046%).

### Data collection and ethical considerations

The protocol of the study adhered to the principles of the Declaration of Helsinki and has been granted administrative clearance for the ethical conduct of research by the host department of the University of the researchers. Our data was collected through a Google Forms survey. Upon approval, we created a Facebook Page where we posted the survey link, together with a poster that indicated a message of invitation and inclusion criteria. For greater reach, we boosted the post and targeted the accounts geolocated in the Philippines and with the age range of interest. The survey collected responses during the first 3 weeks of August 2021. Informed consent was secured digitally. The first page of the online survey presented the details of the study, the voluntary nature of participation, and the rights of the respondents. Privacy and confidentiality were observed in handling the data. The access to the digital data was restricted to us, the researchers.

### Measures

For this study, the predictor variables of interest are gender, digital profile, and social media competence, while the outcome variable is social media political participation in gender issues.

### Gender

Two sub-variables were used to describe the gender of the respondents. First, they were asked about their sex assigned at birth (male = 1, female = 0). Second, they were asked about their sexual orientation and gender identity (SOGI). For sexual orientation, they were asked whether they identify as heterosexual, lesbian, gay, bisexual, asexual, queer, and others. For gender identity, they were asked whether they identify as cisgender or transgender. A “prefer not to disclose” option was provided for both sexual orientation and gender identity. For purposes of categorizing SOGI for inferential analysis, those who identify as both cisgender and heterosexual were grouped as “cisheterosexual”; those who identify as lesbian, gay, bisexual transgender, queer, and/or other non-cisheterosexual identities were grouped as “LGBTQ+”; and those who indicated “prefer not to disclose” in either of sexual orientation or gender identity were grouped as “non-disclosed SOGI.”

### Digital profile

Three sub-variables were measured to characterize the digital profile of the respondents. Through a checklist, they were asked about the different social media sites they used (e.g., Facebook, Twitter, Instagram, etc.), and the different gadgets they owned (e.g., smartphones, laptops, tablets, etc.). An option for “others” was provided for each. The ticks for both indicators were added; the sum of checked items, plus those which were included in “others,” were the values for each. The third sub-variable is internet quality, wherein they were asked to rate from 1 (very bad) to 7 (very good).

### Social media competency scale for college students

Developed by Zhu et al. (2020), SMCS-CS is a 28-item scale that measures social media competence through four domains: technical usability, content interpretation, content generation, and anticipatory reflection. It is measured through a five-point Likert scale (1 = strongly disagree; 5 = strongly agree). This scale has been found to have an acceptable factor structure and internal consistency (Cronbach alpha = 0.97). A sample item is “*I can develop original, visual and textual social media content*.”

### Social media political participation scale

Developed by Waeterloos et al. (2021), SMPP is a 21-item scale that measures four types of political engagement: latent, counter, follower, and expressive engagement, each with acceptable Cronbach alpha values (0.80–0.91). The respondent answers through a six-point scale (1 = never; 5 = very often), with the time reference of within the last 6 months. The sociopolitical issue can be specified on the items of this scale. To examine the specific context of the outcome variable of the study present study, “political issues” were replaced with “gender issues,” thus our coining of SMPP-GI. This scale has an acceptable factor structure, item convergent validity, and overall internal consistency (Cronbach alpha = 0.88). A sample item is “*I commented on something concerning gender issues in a way it was publicly visible*.”

### Data analysis procedure

The dataset was rid of unqualified entries and coded accordingly before formal analysis. Mean and standard deviation was used to describe continuous variables and frequency and percentage for categorical variables. Multiple linear regression analysis (enter method) was used to determine the significant predictors of the four types of SMPP-GI. Bootstrapping (*n* = 5,000) was used to address possible non-normality (Pek et al., 2018). The significance level was set at 0.05 level. JASP version 0.14.1 was used for the statistical analyses.

## Results

### Descriptive results

Table 1 shows the descriptive results of the key variables in the study. Majority of the respondents (*N* = 1,090) are female (*n* = 663, 60.826%) and cisheterosexual (*n* = 825, 75.688%). There are 187 (17.156%) respondents who identify as LGBTQ+, while 78 (7.156%) did not disclose their sexual and/or gender identities. In terms of digital profile, the respondents used an average of four different social media sites (μ = 4.017 ± 1.676), two gadgets (μ = 1.979 ± 0.952), and reported having moderate level quality of internet (μ = 4.747 ± 1.198 out of 7).

**Table 1:** Gender, digital profile, social media competence, and political participation in gender issues (*N* = 1,090)^a^.

**Variables**	***n*** **/mean**	**%/** ***SD***
**Sex assigned at birth**		
Male	427	39.174%
Female	663	60.826%
**Sexual orientation and gender identity**		
Cisheterosexual	825	75.688%
LGBTQ+	187	17.156%
Non-disclosed	78	7.156%
**Number of social media sites used**	4.017	1.676
**Number of gadgets owned**	1.979	0.952
**Internet quality rating**	4.747	1.198
**Social media competence^b^**		
Technical usability	4.012	0.827
Content interpretation	4.091	0.823
Content generation	3.629	0.815
Anticipatory reflection	3.791	0.657
**Social media political participation in gender issues^b^**		
Latent engagement	3.095	0.904
Counter engagement	1.353	0.732
Follower engagement	1.952	1.004
Expressive engagement	2.002	0.934

a *Frequency and percentage for categorical variables, Mean and SD for continuous variables*.

b *Low = 1.00–2.33, Moderate = 2.34–3.66, High = 3.67–5.00*.

The respondents of the study reported high social media competence in terms of technical usability (μ = 4.012 ± 0.827), content interpretation (μ = 4.091 ± 0.832), and anticipatory reflection (μ = 3.791 ± 0.657). On the other hand, the respondents demonstrated moderate competence in terms of content generation (μ = 3.629 ± 0.815).

In terms of SMPP-GI, the respondents reported low levels of counter (μ = 1.353 ± 0.732), follower (μ = 1.952 ± 1.004), and expressive (μ = 2.002 ± 0.934) engagement in gender issues. Meanwhile, the respondents exhibited moderate levels of latent engagement (3.095 ± 0.904).

### Multiple linear regression results

Table 2 shows the results of the multiple linear regression tests to determine the significant gender, digital, and social media competence predictors of social media participation in gender issues. Four bootstrapped models (*n* = 5,000) were tested, one for each type of SMPP-GI. Model 1 significantly predicted latent engagement (*F* = 27.263, *p* < 0.001) and explained 20.3% of its variance. Specifically, variables that significantly predicted latent engagement were sex (*B* = −0.278, *p* < 0.001), LGBTQ+ identity (*B* = 0.628, *p* < 0.001), non-disclosed SOGI identity (*B* = 0.417, *p* < 0.001), number of social media sites used (*B* = 0.059, *p* < 0.001), and content generation (*B* = 0.223, *p* < 0.001). Female respondents, with LGBTQ+ or non-disclosed SOGI identity, who use more social media sites and have higher scores in content generation demonstrate higher levels of latent engagement SMPP-GI.

**Table 2:** Multiple regression tests for predictors of SMPP-GI (*N* = 1,090).

Predictors	**Model 1**			**Model 2**			**Model 3**			**Model 4**		
	**Latent engagement**			**Counter engagement**			**Follower engagement**			**Expressive engagement**		
	***r***^2^ **=** **0.203**, ***F*** **=** 27.263^***^			***r***^2^ **=** **0.070**, ***F*** **=** 8.134^***^			***r***^2^ **=** **0.128**, ***F*** **=** 15.861^***^			***r***^2^ **=** **0.120**, ***F*** **=** 14.710^***^		
	**B**	**SE**	***p***	**B**	**SE**	***p***	**B**	**SE**	***p***	**B**	**SE**	***p***
**Gender**												
Sex assigned at birth (male = 1)	−0.278^***^	0.050	<0.001	0.043	0.046	0.333	−0.187 ^**^	0.059	0.002	−0.094	0.057	0.090
**Sexual orientation & gender identity**												
(LGBTQ+ = 1)	0.628^***^	0.073	<0.001	−0.086	0.058	0.151	0.565^***^	0.093	<0.001	0.489^***^	0.080	<0.001
(Non-Disclosed = 1)	0.417^***^	0.084	<0.001	0.182^*^	0.095	0.027	0.327^**^	0.113	0.004	0.324^**^	0.108	0.002
**Digital profile**												
Number of social media sites	0.059^***^	0.017	<0.001	−0.029^*^	0.014	0.041	0.019	0.018	0.296	0.016	0.017	0.349
Number of gadgets owned	0.030	0.029	0.300	0.032	0.027	0.190	0.046	0.035	0.167	0.044	0.031	0.169
Internet quality	0.025	0.023	0.248	−0.019	0.020	0.304	−0.014	0.026	0.578	−0.027	0.025	0.261
**Social media competence**												
Technical Usability	−0.058	0.056	0.312	−0.088	0.045	0.065	−0.125	0.063	0.050	−0.107	0.060	0.075
Content Interpretation	0.033	0.062	0.583	−0.143^**^	0.053	0.006	−0.056	0.071	0.429	−0.065	0.067	0.306
Content Generation	0.223^***^	0.052	<0.001	0.237^***^	0.040	<0.001	0.402^***^	0.052	<0.001	0.398^***^	0.054	<0.001
Anticipatory Reflection	0.081	0.064	0.186	−0.150^**^	0.045	0.005	−0.104	0.068	0.146	−0.161^*^	0.066	0.016

*Bootstrapping based on 5,000 replicates*,

* *p < 0.05*,

** *p < 0.01*,

*** *p < 0.001*.

Counter engagement was significantly predicted by Model 2 (*F* = 8.134, *p* < 0.001) and explained 7% of its variance. Determinants that yielded significant estimates include non-disclosed SOGI identity (*B* = 0.182, *p* = 0.027), number of social media sites (*B* = −0.029, *p* = 0.041), content interpretation (*B* = −0.143, *p* = 0.006), content generation (*B* = 0.237, *p* < 0.001) and anticipatory reflection (*B* = −0.150, *p* = 0.005). Higher counter engagement levels were observed among respondents with non-disclosed SOGI identity, lesser social media sites, content interpretation and anticipatory reflection, and higher content generation.

Model 3 significantly predicted follower engagement (F = 15.861, *p* < 0.001) and explained 12.8% of its variance. Significant predictors include sex (B = −0.187, *p* < 0.002), LGBTQ+ identity (*B* = 0.565, *p* < 0.001), non-disclosed SOGI identity (*B* = 0.327, *p* = 0.004), and content generation (*B* = 0.402, *p* < 0.001). Females with LGBTQ+ and/or non-disclosed SOGI identities with higher scores in content generation demonstrated significantly higher follower engagement.

Finally, Model 4 significantly predicted expressive engagement (*F* = 14.710, *p* < 0.001) and explained 12% of its variance. Specific variables that yielded significant estimates include LGBTQ+ identity (*B* = 0.489, *p* < 0.001), non-disclosed SOGI identity (*B* = 0.324, *p* = 0.002), content generation (*B* = 0.398, *p* < 0.001), and anticipatory reflection (*B* = −0.161, *p* = 0.016). Expressive engagement was observed to be higher among respondents with LGBTQ+ and non-disclosed SOGI identities who have higher levels of content generation, and lower levels of anticipatory reflection.

## Discussion

Our present study sought to determine the predictive relationship between gender, digital profile, and social media competence, and SMPP-GI among young Filipino netizens. Our investigation contributes to online political participation scholarship by testing the potential of social identity and resource perspectives in understanding and predicting SMPP. To our knowledge, this is the first large-scale study conducted in ASEAN that quantitatively examined gender-related political participation among youth in the context of social media, and competencies related to its use. Our findings suggest that college netizens have low to moderate levels of SMPP-GI. Similarly, Bawan et al. (2017) had previously noted low online political and governance participation in a sample of Filipino professional students.

### Gender and SMPP-GI

Our findings suggest that females demonstrate higher latent and follower engagement SMPP-GI, which confirms previous research that women politically engage in gender issues to enact their gender identity (Chante'Tanksley, 2019; Foster et al., 2021). Meanwhile, LGBTQ+ individuals report higher latent, follower, and expressive engagement SMPP-GI. The present results show that social identity truly fosters political engagement, and supports earlier literature that demonstrates immersive involvement in various social media movements among females and/or LGBTQI+, who are detrimentally affected by patriarchal and cisheterosexist structures that maintain gender inequality (Shevtsova, 2017; Stornaiuolo and Thomas, 2017; Liao, 2019; Hou, 2020). These partially confirm the social identity hypothesis on SMPP-GI.

Interestingly, those who chose not to disclose their sexual and/or gender identities demonstrated higher levels in all types of SMPP-GI, which contrasts with previous evidence demonstrating the active engagement of “out” individuals in gender-related activism (Swank and Fahs, 2013). This finding highlights the nuances of how social identity is enacted in political participation, which may not necessarily involve publicizing one's identity. Concealment of one's potentially marginalized SOGI may be a strategy to protect oneself from further stigmatization and othering (Goffman, 2009) when one engages in political activities online. Non-disclosure can influence the reporting of socially polarizing behaviors, as noted in previous research (e.g., Warner et al., 2020).

### Digital profile and SMPP-GI

Our resource model hypothesis specific to digital profile was partially confirmed, as only one of the indicators yielded significance. Number of social media sites used predicted certain types of SMPP-GI. Specifically, college netizens who use more social media sites report higher levels of latent engagement. This finding emphasizes how the increasingly multi-platform nature of activism can enhance visibility and information consumption of social issues, confirming previous evidence among civil society organizations (Albu and Etter, 2018).

On the other hand, our current findings suggest that college netizens who use lesser social media sites are more likely to practice counter engagement in the name of gender issues. Since the nature and audience of each social media site differ (Albu and Etter, 2018), being in only one site can increase the likelihood of forming an “echo chamber” type social network, which has been noted to exhibit destructive behaviors, such as rumor spreading (Choi et al., 2020).

### Social media competence and SMPP-GI

Our hypothesis on social media competence as a resource that facilitates SMPP-GI is partially confirmed as well, with some SMCS-CS domains demonstrating a significant relationship with certain types of political participation. Among the four domains of social media competence, content generation emerged to be a significant positive predictor of all types of SMPP-GI. Previous research has indicated the positive correlation of functional and critical prosumption to democratic tendencies in social media (Tugtekin and Koc, 2020). Many studies on social media political participation on various issues have highlighted the importance of content creation to engage more people in the cause (Yamamoto and Nah, 2018; Yu and Oh, 2018; Zhu et al., 2019; Tugtekin and Koc, 2020; Yamamoto et al., 2020).

Our findings suggest that anticipatory reflection negatively predicted counter engagement SMPP-GI. Related to this, Zhu et al. (2021) suggests that individuals with lower anticipatory reflection have lesser information society responsibility, which could be linked to counter engagement behaviors. Moreover, it is interesting to note that in our present study, college netizens with high anticipatory reflection are less likely to perform expressive engagement SMPP-GI. We suspect that these students who have increased anticipatory reflection, despite their passion for gender issues, could foresee the possible social consequences of being publicly vocal regarding these issues, such as backlash and loss of friendships, especially from those who have differing opinions about gender-related rights. This fear can make them reluctant to openly participating, as indicated in previous studies (Gustafsson, 2012; Chan, 2018).

Lastly, college netizens who report lower levels of content interpretation have higher counter engagement SMPP-GI. Content interpretation has been linked to information literacy (Zhu et al., 2021). Related to this, Tugtekin and Koc (2020) have suggested that individuals with decreased critical consumption of social media have lower democratic tendencies, which we suspect may result in acts of counter engagement.

### Strengths and limitations of the study

Compared to earlier online political participation studies done among students in the Philippines (e.g., Bawan et al., 2017), our study has a larger sample size and a wider reach. However, it must be cautiously noted that ours is a convenient sample, and Visayas and Mindanao Island groups (South of the Philippines) are not adequately represented. Moreover, the cross-sectional design of the study may decrease the generalizability of the results. Some of the models we tested had quite modest explanatory power (7–20%). It must be noted that political participation is fluid and influenced by the gender issues that are existing during the time of data collection. Future studies can replicate the study on a randomized, representative sample, and include other possible explanatory variables based on other perspectives of political participation. Despite these limitations, our study remains to be the first of its kind in the country and can help stimulate further investigation on SMPP regarding gender and other social issues.

### Conclusions and implications

Our study highlights the current state of social media political participation in the context of gender issues among the Filipino youth, which we have noted to be low to moderate. Youths who are members of gender-disadvantaged categories, such as females and LGBTQ+, demonstrate higher engagement in certain types of SMPP-GI. Truly, the personal is political, and those who are affected by gender-based concerns are more likely to be involved in movements related to them. We also highlight the nuanced nature of social identity in the context of political participation, wherein privatizing and concealing one's gender and/or sexual identity (arguably to protect oneself from stigmatization) can potentially promote higher political engagement in gender issues, as seen in the case of our respondents with non-disclosed SOGI. Hence, designing and implementing programs to elicit effective political participation for gender-related causes among the youth must consider all members across the gender and sexuality spectrum, and include initiatives to protect those who choose not to disclose their identities while they meaningfully engage in these online movements. Future studies can further explore these potential intertwined roles of gender identity and performativity in SMPP-GI.

This present study also identified specific digital affordances (number of active social media sites) and social media competencies that can serve as resources to facilitate SMPP-GI. Content generation emerged to be an important competence that bolsters all types of political engagement. We recommend the development of initiatives that build the capability of the youth in creating engaging content for different social media sites. Future studies can explore the influence of specific content generated on SMPP-GI. Also, campaigns to improve information literacy toward better anticipatory reflection and content interpretation may be implemented to translate counter engagement to less abrasive forms of political engagement. We hope the findings inform stakeholders in social media to promote a more critical, informative, inclusive, and healthier woke culture that advocates gender equality.

## Data availability statement

The raw data supporting the conclusions of this article will be made available by the authors, without undue reservation.

## Ethics statement

The study protocol was granted administrative clearance for ethical conduct of research by the Department of Sociology and Behavioral Sciences of DLSU (2021-08-09). The respondents provided their written informed consent to participate in this study. National and local policies on data privacy were observed during data collection and storage.

## Author contributions

JD and BA: conceptualization, methodology, project administration, formal analysis, and writing—original draft. JC: conceptualization, formal analysis, and writing—final draft. All authors contributed to the article and approved the submitted version.

## Funding

Open access publication will be paid through the publication grant of De La Salle University—Science Foundation.

## Conflict of interest

The authors declare that the research was conducted in the absence of any commercial or financial relationships that could be construed as a potential conflict of interest.

## Publisher's note

All claims expressed in this article are solely those of the authors and do not necessarily represent those of their affiliated organizations, or those of the publisher, the editors and the reviewers. Any product that may be evaluated in this article, or claim that may be made by its manufacturer, is not guaranteed or endorsed by the publisher.
